# Dasatinib-induced renal (or chronic) thrombotic microangiopathy in a patient with chronic myeloid leukemia: A case report

**DOI:** 10.1177/2050313X251322621

**Published:** 2025-02-18

**Authors:** Ryan Sabour, Sohrab Kharabaf, Eric Frazier, Matthew Nguyen, Dao Le, Jonathan Zuckerman, Ramy Hanna

**Affiliations:** 1 Division of Nephrology, Hypertension and Transplant Nephrology, Department of Medicine, University of California, Irvine, CA, USA; 2 Department of Pathology and Lab Medicine, David Geffen School of Medicine, University of California, Los Angeles, CA, USA

**Keywords:** Chronic kidney disease, chronic myeloid leukemia, dasatinib, microangiopathy, nephrology, dasatinib, tyrosine kinase inhibitor

## Abstract

Thrombotic microangiopathy encompasses microvascular thrombosis, hemolytic anemia, thrombocytopenia, and end-organ damage. Secondary thrombotic microangiopathy can result from malignancies, autoimmune diseases, or treatments such as tyrosine kinase inhibitors. Dasatinib, a tyrosine kinase inhibitor used in managing chronic myeloid leukemia, has been linked to thrombotic microangiopathy. This report describes a 66-year-old female with chronic myeloid leukemia treated with dasatinib who developed renal-limited thrombotic microangiopathy. Progressive renal dysfunction found in the context of chronic kidney disease prompted extensive lab evaluation and evaluation, with a renal biopsy confirming thrombotic microangiopathy attributed to dasatinib-induced nephrotoxicity. Discontinuation of dasatinib led to a slight improvement in renal function; however, progressive decline necessitated dialysis. This case underscores the diagnostic and therapeutic challenges of dasatinib-induced thrombotic microangiopathy, emphasizing renal biopsy in diagnosis and monitoring. Individualized treatment strategies and further research should be conducted to optimize future outcomes.

## Introduction

Thrombotic microangiopathy (TMA) is characterized by microvascular thrombosis, microangiopathic hemolytic anemia, thrombocytopenia, and subsequent end-organ damage. Well-known examples include thrombotic thrombocytopenic purpura (TTP), atypical hemolytic uremic syndrome (aHUS) or complement-mediated thrombotic microangiopathy (cm-TMA), and Shiga-toxin *E. coli* induced hemolytic uremic syndrome (STEC-HUS). aHUS can occur secondary to systemic conditions such as autoimmunity, organ transplantation, and certain cancers.^1^ Hematological malignancies and treatments such as hematopoietic stem cell transplants (HSCT), total body irradiation (TBI), and tyrosine kinase inhibitors (TKI) are also linked to TMA.^2^

Dasatinib-induced TMA remains a rarely reported phenomenon, with most documented cases presenting as TTP rather than isolated renal-limited TMA. Two notable case reports describe dasatinib-associated TTP leading to severe renal dysfunction, one of which resulted in end-stage renal disease (ESRD) and subsequent renal transplantation.^3,4^ Beyond TMA, other forms of dasatinib-induced nephrotoxicity have been reported, including acute kidney injury (AKI), nephrotic-range proteinuria, and tubular necrosis.^5–12^ Several reports describe patients developing nephrotic syndrome while on dasatinib, characterized by significant proteinuria, hypoalbuminemia, and kidney injury.^6–10^ The underlying mechanisms remain unclear but may involve glomerular endothelial dysfunction, direct podocyte toxicity, and microvascular injury.^11,12^ This case adds to the growing literature by providing evidence for a chronic, renal-limited form of dasatinib-induced TMA, distinct from acute TTP presentations. Given the paucity of existing cases, further research is warranted to establish risk factors, diagnostic markers, and optimal management strategies.

The mechanism driving thrombosis and hemolysis in cancer involves tumor-induced thrombin production, complement cascade activation, and effects from chemotherapeutic or immunotherapeutic agents.^1^ Microvascular thrombi formation leads to endothelial damage, hemolysis, and platelet aggregation causing ischemia in numerous organ systems.^12^ Ischemic damage can thereby result in renal insufficiency and neurologic compromise. However, certain cases lack overt thrombosis, complicating the differentiation between thrombotic and non-thrombotic microangiopathies.^13^ Prompt recognition and treatment are therefore critical in managing clinical outcomes.^14^

The incidence of TMA varies based on etiology, with secondary TMA representing a majority of cases. Pregnancy, infections, medications, organ transplants, and malignancy are among the most common causes.^15,16^ In hematological malignancies, particularly with HSCT or TKI therapy, TMA risk increases. Reports indicate HSCT-associated TMA incidence of 10%–39% with mortality rates as high as 75%.^17,18^ TKIs compound this risk by altering endothelial proliferation and causing renal dysfunction.^19^ Patients presenting with a combination of these factors face increased risk due to complex physiologic interactions.

In this case report we present a 66-year-old patient with a complicated past medical history of chronic myeloid leukemia (CML), autologous HSCT with TBI therapy, CKD stage IV, and dasatinib treatment who presented to our clinic with worsening kidney function in the setting of chronic kidney disease. This case is unique in that this patient’s renal dysfunction is complicated by a multitude of risk factors for renal dysfunction and TMA making it challenging to distinguish the specific etiology. In this report, we will highlight our approach to evaluation, diagnosis, and management of a unique and complex patient.

## Case presentation

A 66-year-old female with CML in molecular remission, CKD stage IV, hypertension (HTN), and chronic anemia presented for worsening kidney function. Her creatinine, previously stable at 1.6 to 2.0 mg/dL for approximately 10 years before presentation, progressively increased over the past 3 years to 2.5 mg/dL. Initial laboratory evaluation revealed new-onset proteinuria (albumin-to-creatinine ratio 76.3; 24-h urine protein 131 mg/day) without systemic symptoms including peripheral edema, skin rashes, or neurologic deficits. Serum albumin at presentation was 3.9 g/dL, which is above the threshold for hypoalbuminemia (<3.0 g/dL). The patient did not meet nephrotic syndrome criteria, as her urinary protein excretion (131 mg/day) was well below the nephrotic range (>3.5 g/day), and she did not exhibit hypoalbuminemia, peripheral edema, or hyperlipidemia. Blood pressure remained suboptimally controlled (BP > 140/80) despite a regimen of nifedipine, metoprolol, and verapamil.

The patient’s medical history included an autologous HSCT 20 years prior, which was performed despite its limited use for CML, as the patient was in the chronic phase and not a candidate for allogeneic HSCT. The decision to pursue autologous HSCT was influenced by the absence of a matched donor and the need to avoid graft-versus-host disease (GVHD). At the time (~25 years ago), autologous HSCT was occasionally utilized for chronic-phase CML with the goal of deepening remission and potentially eradicating leukemic clones. The patient initially achieved disease control with imatinib, but pre-HSCT treatment included interferon as well. The transplant did not fully eradicate CML, as molecularly detectable disease persisted, necessitating ongoing TKI therapy.

As part of her HSCT conditioning regimen, the patient received TBI at a dose of 1200 cGy, which likely contributed to chronic renal vascular damage. The patient initially achieved disease control with imatinib, but resistance led to the initiation of dasatinib about 12 years prior to presentation. BCR-ABL molecular monitoring showed undetectable levels until 2019 when a 6-week treatment interruption attributed to patient compliance led to relapse (BCR-ABL1: 0.9609% IS). Resumption of dasatinib restored molecular remission (BCR-ABL1: 0.023% IS), which has been maintained since.

Given her ongoing renal decline of unclear etiology, a nephrologic workup, including renal ultrasound, was performed. Ultrasonography demonstrated bilateral echogenic kidneys without hydronephrosis, consistent with chronic parenchymal disease. Serologic workup revealed normal complement levels (C3, C4) and an ADAMTS13 activity of 76%, excluding TTP and complement-mediated thrombotic microangiopathy (aHUS). Infectious and autoimmune causes were also ruled out. In the absence of hydronephrosis, long-standing HTN or TKI (dasatinib)-induced kidney injury were considered the most likely etiologies of acute or chronic renal dysfunction. Given the accelerated renal decline and temporal association with dasatinib therapy, a renal biopsy was pursued in conjunction with increasing the dosage of nifedipine and temporary discontinuation of dasatinib per further workup. A conservative approach was considered as an alternative to biopsy, but it was felt reversibility of drug toxicity would be low if causative. In addition, empiric treatment without firm knowledge of the underlying disease risked potential drug toxicities.

**Figure 1.**
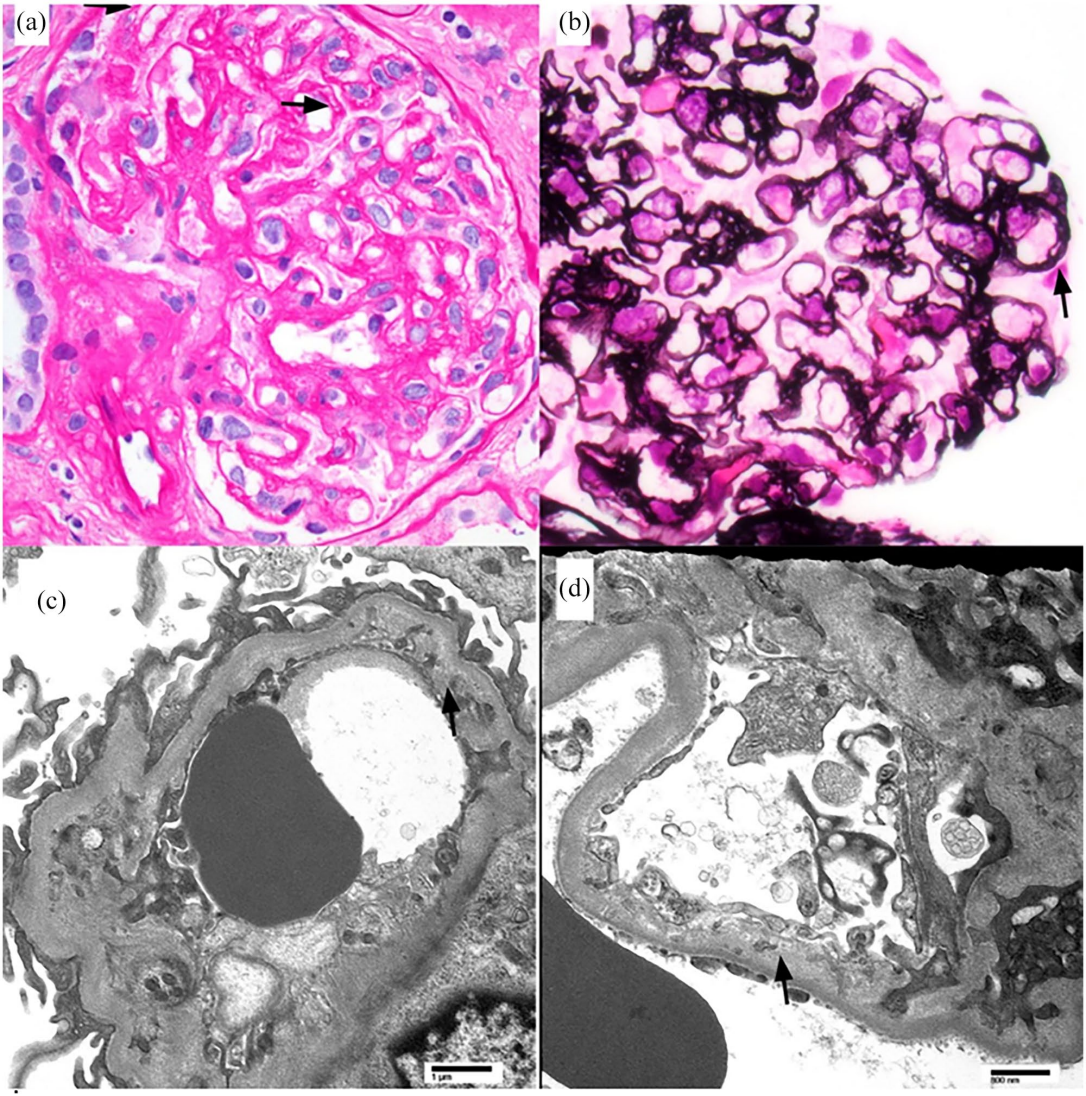
Histologic and ultrastructural findings of chronic thrombotic microangiopathy are observed in renal biopsy . Images from the kidney biopsy demonstrating features of chronic microangiopathy. (a) Light micrograph demonstrating a glomerulus with mild ischemic capillary wall corrugation and segmental capillary wall double contours (arrow) (periodic acid Schiff stain, original magnification 400×). (b) Light micrograph of a glomerulus with segmental capillary wall double contour formation (arrow) (Jones silver stain, original magnification 400×). (c and d) Electron micrographs of glomerular capillaries with widened subendothelial spaces with cellular interposition and deposition of neo-membrane under endothelial cells.

The renal biopsy provided significant evidence supporting a diagnosis of TMA (Figure 1). Chronic microangiopathy was evident through capillary loop double contours, mesangial sclerosis, and segmental glomerulosclerosis, all secondary to chronic vascular injury. Segmental endotheliosis was also evident of a low-grade, ongoing endothelial injury. Electron microscopy revealed subendothelial widening with cellular interposition and new basement membrane formation, consistent with chronic endothelial remodeling. Segmental endothelial swelling further indicated ongoing endothelial stress. Light microscopy demonstrated duplication of the internal elastic lamina in arteries and medial hypertrophy with severe intimal hyalinosis in arterioles, reflecting chronic vascular injury. While no vascular thrombosis was observed, chronic features strongly suggest prior endothelial injury consistent with TMA.

Several findings helped rule out alternative etiologies. Immunofluorescence demonstrated nonspecific staining for IgM, C1q, and C3 in areas of sclerosis, without significant immune complex deposition. Congo red staining was also negative for amyloid deposition. In addition, no significant active inflammatory processes, such as endocapillary hypercellularity or crescents, were present. Systemic causes were excluded by unremarkable complement, ADAMTS13, infectious, and autoimmune workup. HTN, prior TBI, and CKD progression were also considered but were less likely, given the patient’s stable creatinine levels (1.6–2.0 mg/dL) before dasatinib initiation. The temporal correlation between dasatinib use and renal decline, coupled with partial improvement after dose reduction, further supported dasatinib-induced TMA. This was attributed to dasatinib-induced nephrotoxicity and residual sclerosing effects of prior radiation therapy. Given the suspected role of dasatinib in her renal deterioration, we discontinued its usage. To balance CML management, BCR-ABL levels were surveyed regularly with a focus on renal preservation at this time. This initial approach aimed to mitigate kidney damage while assessing the stability of her hematologic condition.

**Figure 2.**
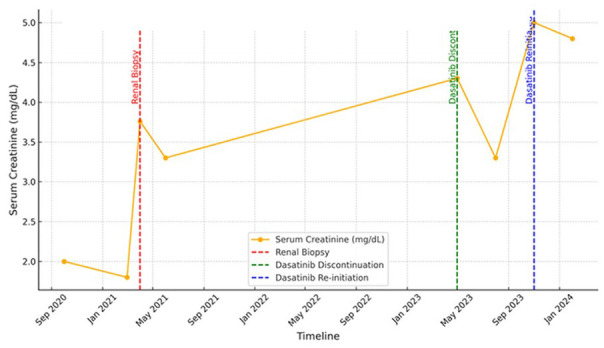
Serum creatinine trends before and after dasatinib therapy. This figure shows the progression of serum creatinine levels (mg/dL) in a patient undergoing dasatinib therapy for chronic myeloid leukemia. Key clinical interventions, including renal biopsy (April 2021), dasatinib discontinuation (May 2023), and re-initiation (November 2023), are marked with dashed vertical lines. The transient improvement in renal function following drug cessation highlights its impact on kidney injury. Data points represent measurements taken at specific time intervals.

In the months post-discontinuation, her creatinine levels showed a slight improvement from 3.76 to 3.3 mg/dL (Figure 2), suggesting some reversibility in her renal function. As a precaution, alternative therapeutic strategies were considered to further prevent kidney decline, including complement blockade with eculizumab. Given the potential for complement-mediated injury in cases of TMA, eculizumab was chosen as it targets the complement cascade, potentially stabilizing renal function.

Despite these interventions, her estimated glomerular filtration rate (eGFR) continued its gradual decline eventually reaching kidney failure at present. Consequently, an arteriovenous (AV) fistula was created in anticipation of potential future dialysis. Dasatinib therapy was restarted in light of kidney failure with dialysis as the mainstay of treatment.

After reinitiating dasatinib therapy, the renal system displayed significant stress just 2 months after with an elevated serum creatinine of 4.3 mg/dL. Her most recent labs approximately 10 months after reinitiating dasatinib therapy show a notably elevated creatinine of 5.5 mg/dL, proteinuria with 2+ urine protein and 143 mg/dL total protein, an elevated haptoglobin of 269 mg/dL, and an elevated LDH of 262 U/L. In addition, her CBC showed decreased RBCs, hemoglobin, and hematocrit at 2.88 million/μL, 9.2 g/dL, and 27.4%, respectively. The dasatinib treatment is being continued on a low dose as it was deemed the best option for her care, however, she continues to receive dialysis and her kidney function is monitored closely. This complex presentation highlights the relationship between dasatinib therapy and progressive renal dysfunction, prompting a broader discussion on the challenges of diagnosis and management of TMA in cancer patients.

## Discussion

Diagnosing and managing TMA in cancer involves differentiation between subtypes (TTP, aHUS, and STEC-HUS). Patients often present with hemolytic anemia, thrombocytopenia, elevated LDH and bilirubin, low haptoglobin, and schistocytes. Drug-induced TMA, such as dasatinib in this case, requires ADAMTS13 levels and direct antiglobulin tests to rule out TTP or autoimmune hemolysis.^12,13,20^ Complement testing, metabolic panels, and renal biopsy add clarity. Biopsies will typically demonstrate mesangiolysis, thickened capillary walls, loss of endothelial cells, occlusion of capillaries, and characteristic deposition of complement C4 proteins in arterioles.^21–23^

TMA treatment involves supportive care, removing the offending agent, and addressing contributing conditions such as HTN. Complement cascade blockade with eculizumab has shown promise^17,18,24^ though plasma exchange therapy (TPE) shows mixed results and any complicated eculizumab dosing.^23^ Consequently, management of TMA is complex and should be individualized based on evaluation and specific etiologies.

This case highlights a rare presentation of limited TMA in a patient with a complex history of CML managed with hematopoietic stem cell transplant and maintenance treatment with dasatinib. Although TMA has been documented in TKI therapy, this case uniquely demonstrates the heightened risk and clinical complexity of renal-limited TMA under combined treatment factors (dasatinib therapy for leukemia control and simultaneous renal preservation).

In contrast to most reported cases of dasatinib-induced TMA where early intervention via drug cessation led to partial or full reversibility of renal dysfunction, this patient’s recovery proved less dramatic. Our patients’ transient improvement was likely indicative of dasatinib’s adverse effects; however, long-term deterioration was attributed to cumulative insult from both dasatinib, prior radiation therapy, and residual effects of HSCT in conjunction with chronic renal disease.

The diagnostic complexity of this case emphasizes the importance of differentiating the variant etiologies of TMA, each with distinct treatment implications and underlying mechanisms. In this case, the patient’s history of CML and treatment with dasatinib, a known TKI associated with TMA, raised initial suspicion. The absence of systemic symptoms (neurologic deficits, GI symptoms) commonly seen in TTP or other TMA supported a limited renal TMA diagnosis in addition to negative lab findings (negative ADAMTS13 levels and complement testing). Ultimately, the renal biopsy proved crucial in confirming TMA-related features such as mesangial sclerosis and segmental glomerulosclerosis, disfavoring non-TMA etiologies initially suspected such as long-standing HTN (Figure 1).

While dasatinib was implicated as a causative factor in the development of TMA, discontinuing or substituting it with an alternative TKI posed significant challenges in maintaining molecular remission. Imatinib, the first TKI trial failed to achieve adequate molecular suppression. Alternative TKIs such as nilotinib and ponatinib were considered but deemed inappropriate due to their known nephrotoxic and vascular toxicity profiles. Given the limited efficacy of other TKIs and the risk of CML relapse, dasatinib was continued at a reduced dosing schedule of 5 days per week. The patient’s renal function was closely monitored, and dialysis was initiated when CKD progressed to ESRD.

Ultimately, a comprehensive workup enabled identification of TKI as the likely causative agent of acute renal dysfunction enabling discontinuation of the offending drug and functional improvement. However, this case raises critical questions regarding the optimal management of TMA in high-risk patients with hematologic malignancies. While eculizumab may stabilize renal function in complement-mediated cases, its role in this patient’s treatment is unclear with no measurable evidence of improving her renal function. However, given this patient’s chronically progressive renal dysfunction, it is impossible to accurately gauge the long-term effects of either dasatinib discontinuation or eculizumab utilization on nephrologic outcomes as a major limitation in this case. Regardless, this case underscores the necessity for individualized treatment in complex TMA cases illustrating the importance of a thorough evaluation and diagnostic workup.

Several limitations must be acknowledged for this case report. Namely, reliance on renal biopsy neither definitively distinguishes TMA subtypes nor confirms causality. The patient’s complex history (radiation and HSCT) further obscures the contribution of dasatinib to renal dysfunction. While this case may offer insights into TMA management, additional research is required to refine protocols and prognostic markers.

## Conclusion

This case highlights the diagnostic and therapeutic challenges in managing TMA in a complex patient. Multiple risk factors, including CML, prior radiation therapy, HSCT, CKD, and TKI therapy complicated the clinical course and obscured etiology. Renal biopsy was instrumental in diagnosing TMA and guiding initial management, but limited renal recovery highlights the multifactorial nature of her disease and current therapeutic limitations.

This case emphasizes the need for a meticulous, individualized evaluation in TMA, particularly those on TKIs where drug cessation might provide only partial reversibility. In addition, the uncertain role and impact of complement blockade therapies such as eculizumab demonstrate the necessity for further research to refine diagnostic criteria, therapeutic interventions, and prognostic markers. Ultimately, this report adds to the growing body of literature on TMA and highlights the importance of multidisciplinary care to optimize outcomes for patients with such complex presentations.

## References

[1] Kavanagh D Goodship TH Richards A . Atypical hemolytic uremic syndrome. Semin Nephrol 2013; 33(6): 508–530. 24161037 10.1016/j.semnephrol.2013.08.003 PMC3863953

[2] Meri S Bunjes D Cofiell R , et al. The role of complement in HSCT-TMA: basic science to clinical practice. Adv Ther 2022; 39(9): 3896–3915. 35781192 10.1007/s12325-022-02184-4 PMC9402756

[3] Martino S Daguindau E Ferrand C , et al. A successful renal transplantation for renal failure after dasatinib-induced thrombotic thrombocytopenic purpura in a patient with imatinib-resistant chronic myelogenous leukaemia on nilotinib. Leuk Res Rep 2013; 2(1): 29–31. 24371772 10.1016/j.lrr.2013.02.003 PMC3850373

[4] Demirsoy ET Mehtap O Atesoglu EB , et al. Dasatinib-induced immune mediated-thrombotic thrombocytopenic purpura. Transfus Apher Sci. 2018; 57(2): 222–224. 29475747 10.1016/j.transci.2018.02.003

[5] Ozkurt S Temiz G Acikalin MF , et al. Acute renal failure under dasatinib therapy. Ren Fail. 2010; 32(1): 147–149. 20113282 10.3109/08860220903391226

[6] Wallace E Lyndon W Chumley P , et al. Dasatinib-induced nephrotic-range proteinuria. Am J Kidney Dis 2013; 61(6): 1026–1031. 23540262 10.1053/j.ajkd.2013.01.022

[7] Ruebner RL Copelovitch L Evageliou NF , et al. Nephrotic syndrome associated with tyrosine kinase inhibitors for pediatric malignancy: case series and review of the literature. Pediatr Nephrol 2014; 29(5): 863–869. 24310825 10.1007/s00467-013-2696-0

[8] Hirano T Hashimoto M Korogi Y , et al. Dasatinib-induced nephrotic syndrome. Leuk Lymphoma 2016; 57(3): 726–727. 26436329 10.3109/10428194.2015.1075020

[9] Lim YT Kim YJ Park YH , et al. A case of dasatinib-induced nephrotic syndrome in a child with philadelphia chromosome positive acute lymphoblastic leukemia. Yonsei Med J 2016; 57(2): 532–533. 26847312 10.3349/ymj.2016.57.2.532 PMC4740552

[10] Ochiai S Sato Y Minakawa A , et al. Dasatinib-induced nephrotic syndrome in a patient with chronic myelogenous leukemia: a case report. BMC Nephrol 2019; 20(1): 87. 30845905 10.1186/s12882-019-1273-6 PMC6407224

[11] Koinuma K Sakairi T Watanabe Y , et al. A case of long-term dasatinib-induced proteinuria and glomerular injury. CEN Case Rep 2020; 9(4): 359–364. 32388829 10.1007/s13730-020-00484-8 PMC7502092

[12] Genest DS Patriquin CJ Licht C , et al. Renal thrombotic microangiopathy: a review. Am J Kidney Dis 2023; 81(5): 591–605. 36509342 10.1053/j.ajkd.2022.10.014

[13] Goodship TH Cook HT Fakhouri F , et al. Atypical hemolytic uremic syndrome and C3 glomerulopathy: conclusions from a “Kidney Disease: Improving Global Outcomes” (KDIGO) Controversies Conference. Kidney Int 2017; 91(3): 539–551. 27989322 10.1016/j.kint.2016.10.005

[14] Werion A Storms P Zizi Y , et al. Epidemiology, outcomes, and complement gene variants in secondary thrombotic microangiopathies. Clin J Am Soc Nephrol 2023; 18(7): 881–891. 37094330 10.2215/CJN.0000000000000182 PMC10356144

[15] Bayer G von Tokarski F Thoreau B , et al. Etiology and outcomes of thrombotic microangiopathies. Clin J Am Soc Nephrol 2019; 14(4): 557–566. 30862697 10.2215/CJN.11470918 PMC6450353

[16] Thomas MR Scully M . Microangiopathy in cancer: causes, consequences, and management. Cancer Treat Res 2019; 179: 151–158. 31317486 10.1007/978-3-030-20315-3_10

[17] Jodele S Laskin BL Dandoy CE , et al. A new paradigm: diagnosis and management of HSCT-associated thrombotic microangiopathy as multi-system endothelial injury. Blood Rev 2015; 29(3): 191–204. 25483393 10.1016/j.blre.2014.11.001 PMC4659438

[18] Brocklebank V Kavanagh D . Complement C5-inhibiting therapy for the thrombotic microangiopathies: accumulating evidence, but not a panacea. Clin Kidney J 2017; 10(5): 600–624. 28980670 10.1093/ckj/sfx081 PMC5622895

[19] Wallace E Lyndon W Chumley P , et al. Dasatinib-induced nephrotic- range proteinuria. Am J Kidney Dis 2013; 61(6): 1026–1031. 23540262 10.1053/j.ajkd.2013.01.022

[20] Mannucci PM Cugno M . The complex differential diagnosis between thrombotic thrombocytopenic purpura and the atypical hemolytic uremic syndrome: Laboratory weapons and their impact on treatment choice and monitoring. Thromb Res 2015; 136(5): 851–854. 26386489 10.1016/j.thromres.2015.09.007

[21] French DL Pollock RR Aguila HL , et al. The molecular and biochemical characterization of mutant monoclonal antibodies with increased antigen binding. J Immunol 1991; 146(6): 2010–2016. 1672339

[22] Laskin BL Maisel J Goebel J , et al. Renal arteriolar C4d deposition: a novel characteristic of hematopoietic stem cell transplantation-associated thrombotic microangiopathy. Transplantation 2013; 96(2): 217–223. 23698598 10.1097/TP.0b013e31829807aa PMC5654605

[23] Laskin BL Goebel J Davies SM , et al. Small vessels, big trouble in the kidneys and beyond: hematopoietic stem cell transplantation-associated thrombotic microangiopathy. Blood 2011; 118(6): 1452–1462. 21596850 10.1182/blood-2011-02-321315

[24] Khosla J Yeh AC Spitzer TR , et al. Hematopoietic stem cell transplant-associated thrombotic microangiopathy: current paradigm and novel therapies. Bone Marrow Transplant 2018; 53(2): 129–137. 28967899 10.1038/bmt.2017.207

